# Book Review

**Published:** 1894-12

**Authors:** 


*A Descriptive Catalogue of the Clinical Museum and Journal of Proceedings.* Part I. By JONATHAN HUTCHINSON, F.R.S., LL.D. Pp. 128. London: J. & A. CHURCHILL. [N.D.]

Jonathan Hutchinson has done as much as any living man for the clinical study of disease; in the establishment of a Clinical Museum, of which the above is a part catalogue, he has secured that his work will have a wider reach and a greater value even than his writings and his lectures.

The purpose of the museum cannot be better described than in Mr. Hutchinson's own words:—

"The Institution which I have ventured to name a Clinical Museum is to some extent an experiment. It is an attempt to show that pictorial representations of disease may be of great use in advancing our knowledge of it, and next to ascertain what are the best methods of displaying them. It is not a new idea. The value of drawings, &c., has been recognised from the earliest times of clinical study, and museum makers from Aldrovandus to Hunter, and from Hunter onwards, have availed themselves of the artist's pencil in order to perpetuate what could not be otherwise preserved. Many atlases of pathology and of clinical surgery, more especially in reference to skin diseases and syphilis, have from time to time been published. A certain number of models and casts, with even a sprinkling of drawings, have also found their way into our general museums of pathology. With, however, the single exception of the splendid collection at the Hôpital St. Louis in Paris, I am not aware that any attempt has yet been made to illustrate systematically the appearances which disease presents in the living subject. I had at one time hoped that this work would have been carried out on a suitable scale in our own College of Surgeons. In connection with the Erasmus Wilson bequest, and the recent enlargements of the Museum buildings, there seemed good reason to believe that this hope was about to be realised. In this belief I even ventured to offer the whole of my own collection for the acceptance of the College, appending, however, to my offer the condition that it should be adequately displayed and not put away in drawers or portfolios. As the Council did not see its way to engage that this condition should be fulfilled, the proposal fell through. The College has, however, devoted one of the galleries to the display of drawings and casts, and of these it possesses a very valuable collection, not a few of the original drawings being unique. Having been forced to realise, however, that it was not probable that during my lifetime anything would be attempted at the College on the plan which I had contemplated, and several other schemes for conjoint enterprise having also come to nothing, I was compelled to fall back on myself and reluctantly to entertain the idea of building a gallery of my own. I had felt that my life was slipping away, and that I could not hope to have many years more in which to work. My collection of drawings, &c., had become large, and from the nature of the materials it would be comparatively useless should it pass into other hands without having been arranged and catalogued by myself. After several disappointments I was fortunate enough to secure the lease of a house behind which there was room for building, and during the summer of 1893 the galleries which now constitute the museum (211 Great Portland Street) were being built. They were opened in November, 1893. Although I was obliged very soon to realise that the space was much too limited, yet there has been reason to be fairly satisfied with the experiment as far as it has gone.

"The design of the Clinical Museum is to collect, from all sources, delineations of diseased appearances in the living subject, and of pathological specimens in their recent state, and to display them in such a way as to be easily accessible to the visitor. It would be absurd to relegate the bones and bottle-specimens which constitute the chief contents of our pathological collections to locked-up cupboards, and it is not less so to keep our drawings stowed away in portfolios. The needs of the present day are to be consulted by making everything as accessible as possible, and especially is display advisable in the case of pictures, of which one main value is that they strike the eye of the observer, and teach their lesson at a glance."

If the purpose of a museum of disease is to show the progress and effects of disease, then is Mr. Hutchinson's one of the most valuable in existence. Nearly all our museums are continued on the assumption that disease is best seen in spirit through the medium of glass. Now "pickle specimens" preserve the form imperfectly, and the colour not at all. They have their value; bleached and distorted as they are, they are still "the thing itself," and they cannot be dispensed with. But they are not the only, and for many diseases they are not the best, means of demonstrating morbid processes. For many, perhaps most, diseases the pickle-jar is not available at all; it is available only for fatal diseases where the specimen is recovered *post mortem*, or for surgical diseases where the peccant portion is removed by operation. For other diseases, the gross termination as shewn in the jar is very different from the varied appearances it exhibited under treatment. Still the pickle-jar remains the mausoleum of pathology; and the orthodox worshipper of the science must make it his resort. The fane, however, is not much frequented. Time after time may one traverse the most magnificent pathological museum in these islands—the Hunterian,—and find oneself the only visitor. The truth of the matter is that we have too much of the pickle-jar. If its appreciation is neglect, it is no more than it deserves.

The simple words in which Mr. Hutchinson refers to the rejection of the offer of his museum to the College of Surgeons are almost pathetic. That it would have added to the value of the Hunterian Museum, the appreciation it has already received is sufficient testimony. That Hunter himself would eagerly have accepted such a gift there can be no doubt. Its permanence ought to be secured on a basis broader than the life of one man, however generous and public-spirited. And the lines on which it has been built up ought to be followed and amplified by those whose business it is to diffuse a knowledge of the external features of disease.

We have been able to make but one visit to the museum, and that only of three hours' duration. In this time it was scarcely possible to get more than a general idea of the grouping and arrangement of the contents. As a sort of test, a small group of diseases in which we were interested was selected, and a cabinet was overhauled. There were some half-dozen drawers in the cabinet, and two hours were spent in superficially examining the contents of two of them. We unhesitatingly affirm that in those two hours we had presented to us a richness and variety of illustration of the diseases in question which no other similar museum collection in Great Britain could match or even approach. It seemed to us as if the clinical student, the pathologist, or the author desirous of bringing to bear on his subject the accumulated knowledge of the past and of vivifying his own conceptions of the disease, were bound to have recourse to the Hutchinson Museum. We can give it no higher praise than this.

In the meantime we may lay the lesson this museum teaches to heart, and consider the duty we, who see disease and pass it through our hands, owe to our successors and our pupils. If one man in the spare moments of a busy lifetime has been able to do so much, what might the properly directed efforts of a whole college numbering its members by the thousand, and reckoning its revenues by tens of thousands not achieve? We might at least hope that the aspirations of our pathologists may soon, with such an example as the Hutchinson Museum before them, lead them beyond their present apotheosis of the pickle-jar.

The Hutchinson Museum may be seen at any time on special application. On Tuesdays from two to six it is always open. Patients are then present, and demonstrations are given often by the medical attendants under whose care they have been.

*Annual of the Universal Medical Sciences.* Edited by Charles E. Sajous, M.D. Five volumes. 1894. Philadelphia: The F. A. Davis Company. London: F. J. Rebman.—Dr. Sajous and his seventy associates, assisted by over two hundred corresponding editors, collaborators, and correspondents, have again brought to a successful issue this work, which is above all criticism. The editorial staff deserve the thanks of the medical world for their continued efforts. We are a little alarmed at seeing some advertisements in one of the volumes, and we hope that they will be kept out of future issues.

*Travaux d'Electrothérapie Gynécologique.* Volume I. Fascicules I. et II. Pp. 720. Paris: Société d'Editions Scientifiques. 1894.—We are exceedingly glad to have this publication on our Exchange list. It is to appear in half-yearly volumes under the editorship of Dr. Apostoli. The use of electricity in gynæcology has not found a large amount of favour in England, and the statements of its results have been so conflicting that we welcome this work, as we believe it will be of the greatest service in permitting an impartial judgment of such methods. The treatment which was introduced by Tripier and improved by Apostoli has been met in some quarters by the most hostile criticism, and the editor in his preface draws attention to the desirability of putting an end to this professional ostracism. He proposes to deal with other than French literature, and he prints, translated into French, contributions of English, American, Russian, German, &c., surgeons, either as original articles or as reprints from the transactions of various societies and medical journals which have appeared in recent years. The work contains a large mass of information on the treatment of all classes of gynæcological disease which have been treated by electricity. The Russian paper is particularly complete. Some of the articles are followed by a summary of the treatment and its results, and we think it would be well if this plan was adopted in all, for the number of cases quoted in detail, although reflecting the greatest credit on the editor and his collaborators, makes the work somewhat difficult to grasp as a whole. The completeness with which this new work has been carried out is a matter for congratulation.

*De l'Extraction des Osselets dans l'Otorrhée.* Dr. E. J. MOURE. Pp. 12. Paris—Bordeaux: O. DOIN ET FERET ET FILS. 1894.—This pamphlet consists of a paper read before the Society of Medicine and Surgery of Bordeaux on November 25th, 1893, and contains reports of two successful cases operated on, together with some judicious general remarks upon the operation and the cases most suitable for its adoption. The author's conclusions are distinctly favourable, and he looks upon the operation as a useful and valuable one in certain cases.

*On Seborrhœa.* By JOSEPH FRANK PAYNE, M.D. Pp. 23. London: JOHN BALE & SONS. 1894.—We have read with great interest Dr. Payne's lecture for the London Post-Graduate Course on "Seborrhœa and its Consequences." The subject is a very important one, for seborrhœa forms the initiative outbreak of a very troublesome form of eczema; hence the sooner the condition is recognised by the medical practitioner the better for the patient, as it is a complaint which mostly comes under his care long before a skin specialist has to deal with it. We mean by this statement that if the complaint is understood—and it is to acquaint him with its earliest symptoms that Dr. Payne has given his lecture—very much good may then be done. We can strongly corroborate the association between seborrhœa and boils, which Dr. Payne so clearly points out; and we are very thoroughly convinced that the most scientific treatment of boils we know of has "antisepsis" very emphatically as its basis. The relationship between seborrhœa capitis and seborrhœa corporis is very intimate, and therefore the comparatively new term for the latter process is unquestionably the correct one; but, whether from early association, or because it more graphically word-paints the condition, we must admit we have an admiration for the older term—Lichen circinatus vel circumscriptus. Treat them both by remedies which have "antisepsis" for their object, and it is astonishing how speedily the complaints are relieved and dispersed. We recommend every practitioner to read Dr. Payne's lecture. He will not be troubled with unnecessary details, and he will gain some invaluable hints about treatment.

*On the Use of Opium in India.* Prepared by ERNEST HART, D.C.L. Pp. vi., 31. London: SMITH, ELDER & CO. 1894.—This reprint from the *British Medical Journal* contains a summary of one hundred reports to the Parliamentary Bills Committee from medical officers and civilians in all parts of India. The testimony of the overwhelming majority of these shows: (1) That from whatever side the opium question in India is viewed, any interference with the present state of things is quite uncalled for; and (2) That not only would no benefit arise from meddling with the old custom of natives, but, on the contrary, any attempt in that direction is certain to be followed by the most serious consequences to the economic, sanitary, physical, and moral condition of our Indian fellow-subjects, and could not fail to prove highly dangerous or, indeed, fatal to our rule in India.

*The Treatment of Chronic Diseases of the Heart by Baths and Exercises.* By WILLIAM BEZLY THORNE, M.D. Pp. 24. London: J. & A. CHURCHILL. 1894.—The system which has been elaborated by the brothers August and Theodor Schott is said to give results such as have never been obtained by any pharmaceutical remedies or other hygienic resources. The author has had the opportunity of observing, in one hour, a shrinking of the area of dulness, as measured obliquely a little below the nipple line, of four, and even six, centimetres, and of two and three centimetres in the vertical line.

*Aids to the Diagnosis and Treatment of Diseases of Children (Medical).* By JOHN McCAW, M.D. Pp. 181. London: BAILLIERE, TINDALL & COX. [1893].—The author states in his preface that this small book claims to be nothing more than a compilation, and expresses his obligation to as many as seven authors. As a collection of extracts from the chief books on children's diseases, it may be useful to students; but how "the busy general practitioner," for whom it is also written, will be able, without an index, to avail himself of the materials contained therein, we do not comprehend. The statements in the work are clearly put; but the omissions are so numerous, that we should not be inclined to recommend the book except to beginners.

*Dwelling Houses: their Sanitary Construction and Arrangements.* By W. H. CORFIELD, M.D. Third Edition. Pp. xiv., 125. London: H. K. LEWIS. 1894.—It is satisfactory to note that Professor Corfield's useful and popular treatise has appeared in another edition. So many books of variable merit are now issued upon this subject, that it is well for the householder to possess a manual upon the accuracy and discretion of which he can thoroughly rely.

*De la Filariosis.* Por el Dr. M. FONT Y TORNÉ. Pp. 20. Barcelona: HENRICH Y C$^{A}$. 1894.—This address to the Medical Society of Cataluña is remarkable for the minute and exhaustive manner in which the subject is handled. The history of the Filaria sanguinis hominis is given from its discovery by Demarquay in 1864 to the present time; not forgetting the labours of

Cobbold (1872), Lewis of Calcutta (1872), and Manson of Amoy (1876), in the same field. The microscopical characters are also detailed at great length, and the letterpress is illustrated by excellent diagrams of the Filaria at 110 and 450 diameters. The habits of the little worms are described *in extenso;* from which it appears that they are quiescent in the daytime, but are to be found in all parts of the circulation at night. The causes, prognosis and treatment are likewise discussed with due regard to the latest observations, and the whole is supplemented by a graphic account of a case seen and treated by Dr. Font, which is believed to be the only one in Europe. The patient was thirty-five, and he was first attacked when eighteen by an intense pain in the left shoulder for twenty-four hours, from thence it descended to the left groin, and thence to the scrotum, followed by an abscess on its posterior and left aspect, which, after eight weeks, gave way and discharged pus mingled with semi-coagulated blood. He had as many as twenty of these altogether from time to time, and they were always accompanied by rigors and elevation of temperature. The left thumb was punctured at three p.m., and the blood was examined and gave no result; but, when the operation was repeated at midnight, the Filariæ were at once perceived under the microscope, moving freely in large numbers. The man lived on the coast between Barcelona and the Pyrenees, and had never been out of Spain.

*Atlas of Clinical Medicine.* By Byrom Bramwell, M.D. Vol. II. Part III. Edinburgh: T. & A. Constable. 1893.—The subjects treated in this part of the Atlas are exophthalmic goitre, acromegaly, general exfoliative epidemic dermatitis; and there is also a reprint of the report of a case of unilateral hypertrophy of the face by Prof. D. W. Montgomery, of the University of California. The clinical descriptions are very clear and good. The article on exophthalmic goitre gives a thorough account of all the symptoms of this disease. The author's view is that the primary lesion is in some part of the nervous system; that as a result of this, the thyroid becomes enlarged, and that its increased or perverted secretion leads to widespread secondary disturbances. He lays stress on the value of electrical treatment. In describing acromegaly, a typical case is taken as the text to illustrate the chief symptoms, and the respects in which it deviates from the common type are pointed out. Dr. Bramwell is inclined to consider the headache, vomiting, optic atrophy or neuritis and temporal hemianopsia so generally present as secondary symptoms due to the enlarged pituitary body, and suggests that polyuria, glycosuria and peptonuria when present may have the same origin. He has administered an extract of the pituitary body with improvement of the symptoms in one patient, in another it seemed to do harm. An interesting point for further examination brought out by cases of acromegaly is the question whether the thyroid and pituitary bodies bear a relation to one another as regards their size. Dr. Bramwell also quotes, as bearing on the diagnosis of acromegaly, Souza-Leite's description of hypertrophic pulmonary osteo-arthropathy, and describes a case of acromegaly in a giantess. The article gives an admirable summary of the whole subject, in which nothing of importance is omitted, and will well repay reading. The directions for examination of such cases will also be found most serviceable. The paper on general exfoliative epidemic dermatitis is an abstract of Dr. Savill's work on the subject, and is accompanied by excellent coloured plates of two of his cases. There are also a series of plates illustrative of acromegaly—photographs of patients, and drawings of the hands and feet; photographs of two men affected with exophthalmic goitre, and a beautiful coloured plate representing the facial changes in old age. This part completes the second volume.

*Post-Nasal Growths.* By CHARLES A. PARKER. Pp. vi., 98. London: H. K. LEWIS. 1894.—In this little work, which is largely a reprint from an article in the current number of *St. Bartholomew's Hospital Reports*, the term post-nasal growths is applied only to adenoid vegetations or hypertrophic enlargement of the lymphoid tissue in the naso-pharynx. It contains nothing very new, but presents us in a readable form with all that is at present known about this troublesome form of malady with the best methods of treating it. The author has evidently had considerable experience of this affection at the Hospital for Diseases of the Throat and at St. Bartholomew's Hospital, and he rightly calls attention to the frequency of its occurrence and the importance of promptly dealing with it. There is a fashion in disease as in most other things, and at the present moment perhaps the subject of adenoids in the naso-pharynx is somewhat overdone, but we can recommend the book before us as one of the best guides on the subject.

*Guy's Hospital Reports.* Vol. L. London: J. & A. CHURCHILL. 1894.—The present volume contains no biographical article such as the two which made last year's Reports so interesting, but it includes some good clinical and pathological material, giving evidence of plenty of earnest research and much diligence in making use of the immense resources of Guy's Hospital. About one-fourth of the volume is occupied by a paper on "Diseases of the Duodenum," by Drs. Perry and Shaw, with an appendix containing details of 334 cases of diseases of this portion of the intestine, taken from English sources; the article will form an invaluable source of reference to any future inquirer. Mr. J. H. Targett contributes an able article on "Hydatids in Bone," illustrated by numerous photographs. The substance of this article formed the subject of an interesting demonstration by Mr. Targett at the recent meeting of the British Medical Association in Bristol. Other noteworthy articles are, "One Hundred Cases of Hyperpyrexia," by Dr. J. H. Bryant; "Clinical Observations upon Heart Disease," by Dr. James F. Goodhart; and "Five Cases of Digital Chancres Occurring in Medical Men," by Mr. W. H. A. Jacobson. Accompanying the volume is a general index to the first fifty volumes of these Reports, which will prove a valuable assistance to those who possess a complete series.

*Handbook of Obstetric Nursing.* By FRANCIS W. N. HAULTAIN, M.D., and JAMES HAIG FERGUSON, M.D. Second Edition. Pp. xiii., 243. Edinburgh: YOUNG J. PENTLAND. 1894.—This work is more comprehensive than it need be for the purpose the authors have in view. A good deal of the information is purely for the expectant mother, and many of the conditions dealt with in Chapter X. do not come within the work of the ordinary obstetric nurse, although the information may be of value to her. We are glad to find that the authors do not allow nurses to use vaginal douching, except by order of the doctor. There should have been some reference to concealed *post-partum* hemorrhage, a very dangerous condition easily overlooked, and which may be suspected when there are severe pains in the back, which must not be mistaken for ordinary after-pains. The chapters on the management of the child and on antiseptics are good; but the use of sponges and washable diapers should not be allowed. No mention is made of iodoform. There is nothing better for use after delivery than a twenty-grain vaginal pessary passed once a day for the first week.

*The After-treatment of Cases of Abdominal Section.* By CHRISTOPHER MARTIN, M.B. Pp. 48. London: SIMPKIN, MARSHALL, HAMILTON, KENT & CO. LIMITED. 1894.—This book is a word-for-word reprint of a series of articles which appeared on this subject in the *Birmingham Medical Review* in 1892–93. The methods of treatment are largely those of Mr. Lawson Tait; but the author differs from that surgeon in his estimation of the value of antiseptics. The teachings are sound and based on considerable experience; and those interested in abdominal work can obtain some valuable hints in these pages. An index would have made the work more useful.

*Sprains: their Consequences and Treatment.* By C. W. MANSELL MOULLIN, M.D. Second Edition. Pp. 153. London: H. K. LEWIS. 1894.—The second edition of this useful book has been almost entirely re-written, and its arrangement materially altered, so that the first half is now devoted to a general treatment of sprains, including methods of treatment that are applicable to all sprains alike; while the latter half treats of individual injuries, giving the special and particular methods of treatment which should be adopted in each case. The general observations in the first part are very well expressed, and are evidently based on a careful consideration of the anatomy and pathology of the parts concerned. Due regard is paid to the once neglected subject of over-rested limbs and joints; and great stress is laid upon judicious and careful "bone-setting." The author has a high opinion of massage in the treatment of sprains, and gives full directions for its application in cases of moderate severity in a healthy person a few hours after the accident. In dealing with particular injuries, notice is taken of such diverse affections as Dupuytren's contraction, ganglion of the wrist, flat foot, etc., many of which can hardly be looked upon as sprains. The author regards the affection known as "lawn-tennis leg" as due, not to a rupture of the plantaris tendon, nor to a tearing of any muscular or tendinous structure in the calf, but to a rupture of one of the veins lying between the muscles of the back of the leg, and he advises that the treatment should be exactly that of a ruptured varicose vein. Mention is made of "lawn-tennis elbow" and "riders' strain," but we fail to find any mention of "golf fore-arm" or "cyclists' strain," both of which are commonly met with; we also look in vain for any description of that curious affection known as "Morton's painful foot" or metatarsalgia, which has been so fully described by Professor Morton, of Philadelphia, and other American surgeons, and which is distinctly of the nature of a sprain. Regarded as a whole, the work is a very valuable epitome of our present knowledge of a subject of universal interest and importance.

*The Middlesex Hospital, W.: Reports of the Medical and Surgical Registrars and Pathologist for the year* 1892. London: H. K. Lewis. 1894.—These excellent Reports, as usual, give a complete account of the work of the Hospital in all its departments; they should be often referred to on account of the statistics of diseases and special reports of interesting or exceptional cases contained in them. Both are of much interest and value. We note two cases of Bèri-Bèri, one of intestinal colic simulating angina pectoris, and, in the treatment of a case of myxœdema, the unpleasant and even alarming symptoms which occasionally follow the administration of thyroid extract. On page 45 *caliac abscess* is obviously a misprint for cœliac axis. In general arrangement and printing the volume deserves high praise.

*Wright's Improved Visiting List, 1895.* Bristol: John Wright & Co.—Several improvements have been made in this: the paper is better, cash columns have been added, a pocket for loose papers is in each cover, and the book fastens conveniently. The great feature of this list—that the names are written up only once a month—has been found to answer admirably, and it saves much time. The book fits comfortably in the breast-pocket, and is a most useful and constant companion throughout the year. We think it a distinct advance on any of its predecessors.

